# Heterogeneity thwarts autism explanatory power: A proposal for endophenotypes

**DOI:** 10.3389/fpsyt.2022.947653

**Published:** 2022-12-01

**Authors:** Lynn Waterhouse

**Affiliations:** The College of New Jersey, Ewing Township, NJ, United States

**Keywords:** autism, heterogeneity, diagnosis, paradigm, DSM-5, subgroups, transdiagnostic

## Abstract

Many researchers now believe that autism heterogeneity is likely to include many disorders, but most research is based on samples defined by the DSM-5 Autism Spectrum Disorder (ASD) criteria. However, individuals diagnosed with autism have complex and varied biological causes for their symptoms. Therefore, autism is not a unitary biological entity. And although autism is significantly different from typical development, autism is not a unitary clinical disorder because diagnosed individuals vary in symptom patterns, comorbidities, biomarkers, and gene variants. The DSM-5 ASD criteria were designed to reduce heterogeneity, and there have been many other efforts to reduce autism heterogeneity including using more stringent clinical criteria, dividing autism into low and high functioning groups, creating subgroups, and by studying larger samples. However, to date these efforts have not been successful. Heterogeneity is extensive and remains unexplained, and no autism pathophysiology has been discovered. Most importantly, heterogeneity has hindered the explanatory power of the autism diagnosis to discover drug regimens and effective behavioral treatments. The paper proposes that possible transdiagnostic endophenotypes may reduce autism heterogeneity. Searching for transdiagnostic endophenotypes requires exploring autism symptoms outside of the framework of the DSM-5 autism diagnosis. This paper proposes that researchers relax diagnostic criteria to increase the range of phenotypes to support the search for transdiagnostic endophenotypes. The paper proposes possible candidates for transdiagnostic endophenotypes. These candidates are taken from DSM-5 ASD criteria, from concepts that have resulted from researched theories, and from symptoms that are the result of subtyping. The paper then sketches a possible basis for a future transdiagnostic endophenotypes screening tool that includes symptoms of autism and other neurodevelopmental disorders.

## Introduction: Heterogeneity thwarts autism explanatory power: Can transdiagnostic behavioral endophenotypes help?

Morris et al. (1) stated that psychiatric diagnoses “have been reified—seen as “real entities” —when in reality they are not natural kinds” (p. 2). Autism is not a unitary biological entity or natural kind (2) because individuals diagnosed with autism have many varied and complex biological causes for their symptoms, and vary in symptom patterns, comorbidities, and biomarkers (3–7). Casanova et al. (8) asserted that, “The diagnostic boundaries of the behavioral phenotype that define ASD are fairly broad due to the large variability that is observed in symptom types, onset, and severity. This variability serves as an index of etiological heterogeneity for a group of complex conditions” (p. 1).

Given this heterogeneity, it is unclear whether or not DSM-5 Autism Spectrum Disorder (ASD) (9) is a unitary clinical entity. Lai et al. (10) argued that DSM-5 ASD *was* a unitary clinical disorder because the field had agreed on unifying elements: the diagnostic criteria; increased prevalence; early presentation; clinical assessments; interventions; cognitive processes; and links to multiple causal agents. However, Tunç et al. (11) reported evidence for a fuzzy boundary between ASD and non-ASD that did not result from misdiagnosis, and they stated that an “ASD or a non-ASD diagnosis at a given time should then be seen as a ‘current’ state of a child within the phenotypic and developmental continuum” (p. 1237). Moreover, Hyman (2) argued that no psychiatric diagnostic categories can be unitary entities because they all include heterogeneous symptoms, gene variants, significant comorbidity and varied biomarkers “accompanied by a large variability of other symptoms” (p. 3).

Importantly, heterogeneity has hobbled the explanatory power of the autism diagnosis. In 2021, McCracken et al. (12) reported that “Two decades of increases in intervention research funding with advances in the basic neuroscience understanding of ASD has not produced progress in pharmacological interventions for ASD core deficits” (p. 4). In 2021, the Lancet Commission Report (13) asserted that research has yet to discover behavioral treatments for the “heterogeneity of manifestations of autism” (p. 300). However, because autism heterogeneity is so extensive, if the Lancet Commission Report's goal is finding behavioral treatments for each of the heterogenous “manifestations of autism” then hundreds of studies will be needed to discover unique behavioral treatments for each manifestation of autism. Moreover, Shic et al. (4) noted that “Progress in developing interventions for ASD has been hindered by a lack of measures that can, within this heterogeneity, provide objective quantification of intrinsic features of ASD with sensitivity, reliability, and mechanistic relationship to core symptoms” (p. 2).

Borsboom et al. theorized heterogeneity would be resolved by one large network encompassing all symptoms and causes for all childhood and adult DSM-5 psychiatric disorders (14). By contrast, Wolfers et al. (15) noted that autism should be best understood “at the level of the individual” (p. 250). This paper argues that because autism heterogeneity, including comorbidities, is so extensive, it is likely that transdiagnostic neurodevelopmental social impairment endophenotypes may be found within and across the current diagnostic boundaries of autism and other neurodevelopmental disorders (5, 6, 8, 11, 16, 17).

The paper proposes that transdiagnostic social impairment endophenotypes may address heterogeneity by discovering meaningful transdiagnostic social impairment neurodevelopmental groups. Although social impairment is just one of the two diagnostic criteria for DSM-5 ASD, the other being restrictive/repetitive behaviors (RRB), social impairment has remained the core feature of all those diagnosed with autism, and therefore is a good candidate for establishing concepts that can yield productive transdiagnostic endophenotypes.

Of course, transdiagnostic behavioral social impairment endophenotypes may not exist or may not be of value, but they cannot be proven valueless if researchers continue to study samples defined by the DSM5 ASD diagnosis. As Hyman (2) noted, studying samples based on DSM diagnoses reflects the “dogged persistence of the *DSM* categorical approach, notwithstanding a large, convincing, and still growing body of negative evidence” (p. 21).

This paper has six sections. The first section, Transdiagnostic endophenotypes, (a) defines endophenotypes, (b) provides examples of the transdiagnostic endophenotype approach, and (c) discusses the relationship of transdiagnostic endophenotypes to diagnoses. The second section, The DSM-5 ASD, explains how the DSM-5 spectrum diagnosis (a) was designed to reduce heterogeneity in autism by creating a single diagnostic category, (b) but has excluded many individuals from diagnosis, and (c) still allows a wide range of heterogeneity. The third section, The extent of autism heterogeneity, that occurs along with DSM-5 ASD outlines current evidence for autism heterogeneity. The fourth section, Efforts to reduce autism heterogeneity, outlines current efforts to reduce autism heterogeneity. The fifth section, Finding transdiagnostic endophenotypes, explores possible constructs for transdiagnostic behavioral endophenotypes (a) from DSM-5 ASD and ADOS (18), (b) from theories of the causes for social impairment, and (c) from autism subgrouping. The sixth section, Pro tem sketch for a future transdiagnostic endophenotype symptom screening, proposes sets of autism symptoms and comorbid disorder symptoms that could provide a basis for items on a screening tool to discover transdiagnostic endophenotypes.

## Transdiagnostic endophenotypes

### Definitions of endophenotypes and transdiagnostic endophenotypes

An endophenotype aggregates a group of phenotypes of affected individuals, for which a specific behavioral trait, gene variant or biomarker exists with explanatory power across the affected individuals' diagnostic category. Endophenotypes were first defined as gene variants used to explore behavioral traits or biological markers, but endophenotypes now include using behavioral traits or biological markers to explore possible links between other features of a disorder (19). An autism biomarker endophenotype, such as abnormal brain white matter (20), could be used to search for a narrowed set of gene variants, or narrowed set of symptoms and behaviors. And a behavioral endophenotype can also be used to index other behavioral endophenotypes. For example, The Autism Biomarkers Consortium for Clinical Trials (ABC-CT) is using a behavioral measure, Oculomotor Index of Gaze to Human Faces (OMI), to explore three attention behavior patterns: Activity Monitoring, Social Interactive, and Static Scenes (4).

Transdiagnostic endophenotypes are endophenotypes that explore behavioral traits, gene variants or biomarkers that cross diagnostic boundaries. For example, Rommelse et al. (17) argued for the use of autism gene variant endophenotypes that might link ASD with Attention Deficit Hyperactivity Disorder (ADHD).

### Examples of transdiagnostic endophenotypes

The Research Domain Criteria (RDoC) project (1), initiated by the National Institute of Mental Health, is designed to find transdiagnostic pathophysiologies by means of functional behavioral constructs. RDoC is one of two large projects studying transdiagnostic behavioral endophenotypes in adult disorders, the other is the Psychiatric Ratings using Intermediate Stratified Markers (PRISM) project (20, 21). Both projects use transdiagnostic endophenotypes as devices for exploring the possible shared biological bases of psychiatric disorders and symptoms. Currently, the PRISM project is using the transdiagnostic behavioral endophenotype of social withdrawal as a means to explore brain regions that are impaired across psychiatric disorders.

The ESSENCE model, Early Symptomatic Syndromes Eliciting Neurodevelopmental Clinical Examinations (16), is a transdiagnostic umbrella that addresses the problem that many children with neurodevelopmental diagnoses such as ASD and ADHD have symptoms that cross diagnostic boundaries. Gillberg proposed “There is good evidence that ASD and ADHD can be separate and recognizable ‘disorders’, but, equally, there is mounting evidence that they often overlap, constitute amalgams of problems, and that in some families they separate together and probably represent different aspects of the same underlying disorder” (p. 1544).

Other diagnoses included under the ESSENCE umbrella include Specific Language Impairment, Oppositional Defiant Disorder, Developmental Coordination Disorder, Tic disorders including Tourette syndrome, Bipolar Disorder, behavioral phenotype syndromes, rare epilepsy syndromes, and Reactive Attachment Disorder. The main behaviors of these disorders are impaired motor skills, social interaction, speech and language, attention, sleep, activity levels, and general developmental delay. Gillberg (16) stated that “a reasonable estimate would be that about 5–7% of children under age 6 years would meet ‘criteria’ for ESSENCE (i.e., have clinical symptoms of a syndrome and have presented at a clinic with a view to diagnosis and intervention)” (p. 1545).

### Endophenotypes and diagnostic categories

RDoC, PRISM, and ESSENCE preserve diagnostic boundaries while simultaneously searching for transdiagnostic endophenotypic commonalities across diagnoses. None of these three approaches is designed to form a new diagnosis from an endophenotype. Like RDoC, PRISM, and ESSENCE, the goal of the transdiagnostic endophenotypes proposed here is to discover groups with commonalities across autism and comorbid diagnoses. If, however, a transdiagnostic neurodevelopmental endophenotype of social impairment can identify a group of affected individuals who share a significant number of symptoms and causes, there may be meaningful transdiagnostic social impairment neurodevelopmental diagnostic groups.

## The DSM-5 ASD

### Unifying autism through the DSM-5 ASD diagnosis

Rosen et al. (22) claimed that prior to the DSM-5 ASD diagnosis, research was “a history of largely unsuccessful attempts to categorize the heterogeneity of autism into empirically-defined subcategories” (p. 13), and they argued that having just one DSM-5 autism diagnosis addressed heterogeneity by eliminating these subcategories. The researchers noted that the previous five diagnoses were abandoned because those five disorders [autistic disorder, Asperger's disorder, pervasive developmental disorder not otherwise specified (PDD-NOS), Rett's disorder, and childhood disintegrative disorder] did not have distinct symptom profiles and the five failed to be differentially predictive.

The DSM-5 ASD includes two core diagnostic symptom groups, social deficits and RRBs. These criteria are understood as dimensional, on a continuum of typical to atypical behaviors. These symptoms must be present in early development, and must cause significant impairment in current functioning. Moreover, and when symptoms of ASD and intellectual disability (ID) occur together, social communication should be below that expected for general developmental level. Within social deficits and RRBs there are three specified levels of support (requires support, requires substantial support, and requires very substantial support). Also, in DSM-5 ASD, language is a separate non-diagnostic dimension, defined as a specifier. In DSM-5 ASD impaired conversation became a symptom of impaired social-emotional reciprocity within social deficits, and stereotyped language became a symptom within the RRBs. Vivanti and Messinger (23) asserted the dimensional model moved away from “grand theories focused on autism as a unitary and monolithic entity to the examination of specific phenomena and processes” (p. 13). Grzadzinski et al. (24) claimed that the dimensions approach would reduce heterogeneity by allowing “the identification of subgroups within ASD that will be important for understanding the biological mechanisms, clinical outcomes, and treatment responses” (p. 4).

### Exclusion of individuals from a DSM-5 ASD diagnosis

Although Lai et al. (10) argued, “DSM-5 ASD criteria should be commended” (p. 3) for their effectiveness in diagnosing individuals, Kulage et al. (25) reviewed studies of the effect of DSM-5 criteria on autism diagnoses and reported that “More than half of the studies included in this systematic review and meta-analysis demonstrated ASD reduction rates between 25 and 68% when applying DSM-5 criteria” (p. 1930). Thus, there are now many who previously had an autism diagnosis but do not meet the DSM-5 ASD criteria, and therefore “have fallen outside of DSM-5 thresholds for receiving state-funded, school-supported, and/or insurance-covered services for their developmental, social, and communication deficiencies” (p. 1930).

### DSM-5 ASD heterogeneity

Although DSM-5 ASD reduced the heterogeneity of five diagnostic categories to one, nonetheless the two DSM-5 ASD core diagnostic dimensions, social deficits and RRBs, allow for a wide range of diagnostic symptom patterns. Moreover, the DSM-5 criteria for ASD require the specification of whether ASD occurs with intellectual disability (ID), language impairment, other neurodevelopmental, mental, or behavioral disorder, or with a known medical or genetic condition or environmental factor. Although these specifiers exist outside the core diagnosis, the wide range of specifiers means that there will be heterogeneity in any ASD sample studied.

Wiggins et al. (7) found that one ASD heterogeneity factor—symptoms of dysregulation including anxiety, depression, aggression, and sleep problems—was responsible for 49–65% of the variance in an ASD sample. They also reported that expressive and receptive language skills were responsible for an additional 15–30% of the variance. The presence of sensory dysfunction was the only symptom that defined homogeneity for ASD, and they recommended that sensory dysfunction should be added as a core diagnostic symptom.

## The extent of autism heterogeneity

Researchers have identified heterogeneity in many aspects of autism. Here are some recent studies that report heterogeneity.

### Gene variant heterogeneity

A review of gene variants reported that autism has been found with multiple single nucleotide polymorphisms (SNPs) for all major synapse types, serotonergic, dopaminergic, GABAergic, and glutamatergic, as well as with many copy number variants (CNVs)–deletions or duplications of DNA (26).

### Comorbidity heterogeneity

McCormick et al. reviewed comorbidities in autism and noted that 47% of those with autism had another neurodevelopmental disorder, 44% had a psychiatric disorder, 43% had a neurological condition, and 93% had a medical condition that was not neurological or psychiatric (27).

### Biomarker heterogeneity

Girault et al. (28) noted that there are many varied forms of brain disorder in autism. These include aberrant white matter integrity, aberrant connectivity, altered morphology of the corpus callosum, increased extra-axial CSF volumes, cortical surface area hyperexpansion, and greater total cerebral volume. However, Martinez-Murcia et al. found no difference between neuroimages of individuals with autism and typically developing controls (29). Although many autism biomarkers have been identified (30), no autism-specific set of biomarkers has been found (31). However, The European Autism Interventions - A Multicentre Study for Developing New Medications is currently searching for a comprehensive set of significant biomarkers (32).

### Subgroup heterogeneity

Many subgroups of autism have been proposed (5–8, 16, 17, 24). A wide range of subgroups was discovered in thirty studies from the Autism Phenome Project (33). Across the thirty studies they identified nine endophenotypic subgroups of autism: (a) disproportionate megalocephaly; (b) external hydrocephalus; (c) distress at noise with good cognition; (d) mothers with maternal IgG autoantibodies that bind to fetal brain tissue; (e) significant GI problems; (f) anxiety disorders; (g) IQ variation across development; (h) high levels of atypical sensory behavior; and, (i) higher IQ females with decreasing autism symptoms over time (33).

### Increased heterogeneity generated by increasing prevalence

Changes over time in the autism diagnostic criteria and autism research findings have resulted in an ever-increasing autism prevalence. This increasing prevalence has added to autism heterogeneity. In 1967, Wing et al. reported a U.K regional prevalence of core autism as 2.1 in 10,000 or 0.00021% (34). The U.S. prevalence was recently reported to be 1.85% (35), and a 2022 study of data from the National Health Interview Survey (36) reported that the prevalence of ASD was 2.79% in 2019, 3.49% in 2020 and 3.14% for both years combined. A 2022 U.S. survey found a 2.6% prevalence rate among Spanish-speaking families (37). Saito et al. reported a prevalence of 3.22% for Japan (38), and Schendel et al. reported that a lifetime incidence of autism in a Danish cohort ranged from 3.52 to 4.28% (39). By contrast, schizophrenia prevalence was 0.33% before 1990, and 0.51% after 2013 (40). Thus, the prevalence of schizophrenia was 2,429 times that of autism before 1970, and currently the prevalence of schizophrenia is between one-third and one-sixth that of autism.

### Heterogeneity of the wide range of ASD high and low symptom severity

The severity of autism impairment has changed significantly over time. Wing et al.'s (34) autism criteria combined low functioning which was significant intellectual disability with the absence of speech, along with high functioning which was islands of normal intelligence. Currently, the DSM-5 ASD diagnosis includes severity of symptoms ranging from needing complete support to needing very little or no support. For example, within the DSM-5 ASD diagnosis, there are very high functioning individuals identified as “on the autism spectrum” who can speak their minds and publish articles criticizing the DSM-5 for missing crucial subtypes and having criteria that are outdated (41). And Roman-Urrestarazou et al. (42) have proposed many with autism could be a socio-political force. They argued that “it's time for a change, and we should start by asking autistic people how they would like to be called and recognizing the long civil rights struggle that they have endured to be recognized and validated in their lived-in experience” (p. 634).

However, in contrast to very high functioning individuals with autism who can form a lobbying group, there is evidence for an increased number of low functioning individuals with autism. An analysis of change in impairment levels over time in 27,240 individuals in the Child and Adolescent Twin Study in Sweden found that level of autism impairment increased with consecutively later birth cohorts (43). The authors suggested that the autism diagnosis was expanding and raised doubt that a clinically relevant syndrome was formed by social communication deficits with RRBs.

### Heterogeneity of life course in ASD

Tunç et al. (11) reported that “children with ASD have heterogeneous developmental trajectories” (p. 1236). Steinhausen et al. (44) conducted a meta-analysis of studies of ASD outcomes. They reported that “Across the various studies an estimated percentage of 19.7% demonstrated a good outcome, close to 31.1% had a fair outcome, whereas close to half (47.7%) of the participants had either a poor or even a very poor outcome in adulthood” (p. 450). Elias and Lord (45) studied four outcome groups, Retained ASD, Lost ASD, Never Had ASD and Gained ASD Diagnosis, and the researchers concluded that diagnoses of autism can shift across development.

### Heterogeneity of treatments

A recent report outlined the current lack of effective drug treatment regimens for autism: “Dozens of clinical trials… have so far failed to identify any pharmacologic treatments for the core symptom domains of social deficits and restricted/repetitive behavior” (46). However, a new drug development platform, the Autism Spectrum POC (proof of concept) Initiative (ASPI) (47) is working to create effective treatment regimens. It remains a problem, though, that the ASPI project relies on biomarkers, because a set of significant biomarkers has not yet been established (30–32). And the effectiveness of behavioral interventions remains unclear. Sandbank et al. (48) and Bottema-Beutel et al. (49) reported that studies of behavioral interventions are not sufficiently well-designed, thus we do not yet know the effectiveness of autism behavioral interventions. Moreover, as noted in the Introduction, The Lancet Commission Report (13) asserted that varied behavioral treatments for the “heterogeneity of manifestations of autism” (p. 300) need to be discovered.

## Efforts to reduce autism heterogeneity

In addition to establishing the DSM-5 ASD diagnosis as a means to reduce autism heterogeneity, other ways to reduce autism heterogeneity have been proposed, including the use of more stringent clinical criteria; excluding moderate-to-severe autism symptoms; creating subgroups; and increasing study sample sizes.

### Reducing heterogeneity by determining prototypical autism

Mottron proposed prototypical autism, a new diagnosis that would reduce ASD heterogeneity by requiring two clinicians to agree on a more circumscribed set of autism criteria (50). Mottron posited that in an autism sample homogeneous for comorbidity, language problems, intelligence, age and sex, prototypicality would be determined by two experts based on their clinical knowledge of autism, and their speed of clinical identification. One difficulty for this proposal is that DSM-5 ASD criteria have already significantly reduced the number of affected individuals being diagnosed with autism (25), and prototypical autism would further reduce the number of DSM-5 ASD diagnosed individuals.

### Reducing heterogeneity by dividing levels of functioning

Wiggins et al. found that excluding moderate-to-severe autism symptoms reduced autism heterogeneity (7). Lord et al. created a new administrative classification, profound autism, to better provide care for the lowest functioning individuals (13). This, de facto, formed two categories: “autism-profound” defined by an IQ below 50 and an inability to use comprehensible sentences; and “autism-not profound”.

### Reducing heterogeneity by creating subgroups

As noted above, Grzadzinski et al. (24) claimed that forming subgroups would reduce heterogeneity, and Nordahl et al. outlined nine unique subgroups within autism (33). In addition, many different subgroups of autism have been proposed (51–54) to reduce heterogeneity by finding factors and clusters defining grouping symptoms.

### Reducing heterogeneity by using larger samples

Many researchers have argued that very large sample sizes would reduce heterogeneity. Chen et al. claimed that mammoth data sets would resolve heterogeneity (55), and Vivanti and Messinger (23) argued that studying “vast quantities of behavior” (p. 4316) would explain autism phenotype heterogeneity.

Happé and Frith (56) optimistically predicted, “As sample sizes in autism genetic consortia rise, polygenic scores for autism may begin to explain a meaningful proportion of variance in autistic traits” (p. 224). There are polygenic risk scores for autism, however, as gene study sample size has increased, explaining autism variance has gotten more difficult and not less difficult, because larger samples have revealed more associations with ID, ADHD, anxiety disorders, schizophrenia, and other non-autism disorders and symptoms (57). Moreover, increasingly larger samples have led to the discovery of more CNVs and SNPs that converge on one autism behavior, and to the discovery of subsets of gene variants linked to subsets of autism behaviors (58). Overall, larger samples have increased genetic heterogeneity, and have identified many forms of syndromic autism, but have not untangled gene-behavior causal complexities in idiopathic autism as was predicted by Happé and Frith (56).

Lombardo et al. (59) asserted that, “Small samples cannot adequately cover heterogeneity in the autism population in a highly generalizable fashion, and hence there is a need for ‘big data’ when studying heterogeneity. Big data should be both broad and deep, to not only sample adequately across different strata from the population but also to examine how strata defined at one level may be relevant for explaining variability at other levels” (p. 1446–1447).

### Summary of efforts to reduce heterogeneity

The efforts for reducing autism heterogeneity—creating prototypical autism, excluding moderate-to-severe autism symptoms, creating subgroups, and increasing sample sizes–have not yet been effective in reducing heterogeneity. Thus, heterogeneity remains an unresolved problem (2–17, 26–49). Moreover, as discussed previously, establishing the unitary DSM-5 ASD diagnosis has not reduced autism heterogeneity.

## Finding transdiagnostic endophenotypes

There are two crucial steps for finding transdiagnostic endophenotypes. The first step is to relax DSM-5 ASD criteria in order to increase the range of phenotypes to include many individuals with subthreshold ASD. The second step is to find constructs for endophenotypes in DSM-5 ASD criteria, in theories of autism, and in autism subgroups.

### Step one: Relaxing DSM-5 ASD criteria to create a large sample of phenotypes

Relaxing DSM-5 ASD criteria is the first step toward discovering transdiagnostic endophenotypes because having a large pool of phenotypes that includes those diagnosed with DSM-5 ASD, and those with autism symptoms who do not fully meet the criteria for ASD, provides the best chance for discovering transdiagnostic endophenotypes. For example, relaxing the DSM-5 ASD criteria will likely allow the inclusion of the 25–68% of affected individuals that Kulage et al. (25) found were excluded by the DSM-5 ASD criteria.

Relaxing the DSM-5 ASD criteria will also include affected individuals at the ASD fuzzy boundaries with comorbidities. Wiggins et al. (7) stated that “phenotypic diversity in preschool children with ASD symptoms extends beyond diagnostic boundaries” (p. 548), Tunç et al. (11) reported evidence for a fuzzy boundary between ASD and non-ASD that did not result from misdiagnosis, and Gillberg (16) noted that there are significant overlaps between autism and many other neurodevelopmental disorders. And as noted earlier, there is a very high level of comorbidities for autism: McCormick et al. reported (27) that in those diagnosed with DSM-5 ASD 47% had another neurodevelopmental disorder, 44% had a psychiatric disorder, 43% had a neurological condition, and 93% had a medical condition that was not neurological or psychiatric.

#### The existence of syndromic autism argues for relaxing the DSM-5 ASD criteria

Although research has found many forms of syndromic autism, Waye and Cheng pointed out that no treatments for syndromic autism have been discovered (60). Eighty-five percent of autism is idiopathic, i.e., of unknown cause, and 15% is syndromic autism, for which a cause has been identified. Syndromic autism includes Rett's disorder, tuberous sclerosis, Down syndrome, Fragile X syndrome (FXS), and congenital infections such as cytomegalovirus. Ziats et al. (61) conducted a comprehensive review of syndromic autism and found 180 autism syndromes. Of the 180 syndromes, 59 syndromes were unique to autism and included loci and chromosome duplication and deletion syndromes, six were chromosomal aneuploidy disorders, and 115 were single gene disorders. Notably, in only 17 of the 115 monogenic syndromes did most patients meet DSM autism criteria.

Because many with syndromic autism do not meet the full DSM-5 criteria, and because many with idiopathic autism have links to gene variants, if the DSM-5 criteria were relaxed, then it is likely that many partial idiopathic autism phenotypes would be found. As noted above, this would be of significant value, because having more phenotypes would improve the chance to discover transdiagnostic endophenotypes.

#### Co-occurring autism with ID argues for relaxing autism criteria

ID is variably expressed with autism symptoms. Approximately forty percent of those with autism are non-verbal, and roughly thirty percent of those who can be tested have IQs below 70. Nordahl et al. (33) reported three patterns of ID in autism: a large majority with persistent ID; a minority with no ID; and some with ID improving to the normal range by age 6–7.

Thurm et al. claimed autism and ID are two distinct disorders (62). However, ID is most likely to be evidence of additional symptoms in individuals with autism, and not evidence of an additional comorbid disorder. As Carpenter pointed out, diagnostic divisions are unlikely to divide causes for symptoms (63). ID and autism are unlikely to be separate comorbid disorders, (a) because many of those with syndromic autism express both ID and autism symptoms, and (b) because a large subset of those with idiopathic DSM-5 ASD are diagnosed with ID, and (c) because many ID and autism symptoms overlap.

Thurm et al. argued that it is crucial to determine whether autism or ID is more prominent in an individual. They asserted that “Whereas ID is associated with general deficits across developmental domains, ASD is in fact defined by the observation that social communication deficits *are particularly impairing*” (p. 2). However, ID and autism are not effectively divided by “particularly impairing” social communication because the DSM diagnosis allows for high functioning individuals who hold jobs and write articles and do not have “particularly impairing” social communication deficits (41, 42).

Because ID and autism symptoms occur together, DSM-5 criteria should be relaxed to include individuals who do not meet all DSM-5 criteria, and who may also be diagnosed with ID, ADHD, anxiety disorder, and other disorders. Opening the diagnosis will enrich the number of phenotypes, and thus will provide a greater chance for discovering possible transdiagnostic behavioral endophenotypes.

### Step two: Searching DSM-5 ASD criteria, autism theories and autism subgroups for possible endophenotype constructs

Step two is to search three likely sources for symptom constructs for endophenotypes. The first source is the DSM-5 ASD criteria and the social impairment symptom groups found in diagnostic assessments. The rationale for searching here is that these diagnostic symptoms have documented significant differences between autism and typical development, and these diagnostic symptoms include symptoms that are found in other neurodevelopmental disorders. The second source is the causal autism symptoms proposed in theories of social impairment in autism. The rationale for searching here is that theories propose specific mechanisms that might be the source of social impairment. The third source is the symptoms found in ASD subgrouping studies. The rationale for searching here is that subgrouping studies employ factor analysis and cluster analysis to discover symptoms identified in significant subgroups.

#### The first sources for possible transdiagnostic constructs are DSM-5 ASD and ADOS symptoms

##### *DSM-5 ASD* social impairment symptoms

Because social impairment has been the core autism symptom from past to present, social impairment constructs may be a good place to start the search for possible endophenotypes in autism. DSM-5 social impairment criteria describe both (a) the inability to engage socially, and (b) socially engaged behavior that is impaired. Inability to engage includes making little or inconsistent eye contact; appearing not to look at or listen to people who are talking; and trouble in responding to one's name or to other verbal bids for attention.

Impaired social engagement behaviors include: infrequently sharing interests, emotion, or enjoyment of objects or activities; having difficulties with the back and forth of conversation; often talking at length about a favorite subject without noticing that others are not interested or without giving others a chance to respond; displaying facial expressions, movements, and gestures that do not match what is being said; having an unusual tone of voice that may sound sing-song or flat and robot-like; having trouble understanding another person's point of view or being unable to predict or understand other people's actions; difficulties adjusting behaviors to social situations; and, difficulties sharing in imaginative play.

The simple division of autism diagnostic social impairments into two constructs, *inability to engage socially*, and *impaired socially engaged behavior*, may serve as endophenotypes.

##### *ADOS* social impairment symptoms

A study of ADOS (18, 64) social impairment items by Bishop et al. (65) discovered two subgroups: (1) basic social communication, which included eye contact, facial expression, gesture, and shared enjoyment; and (2) interaction quality, which included the amount of reciprocal social communication, conversation, and overall quality of rapport. Scores for interaction quality, but not for basic social communication, were linked to non-verbal IQ and to being male. The researchers' goal was to predict an autism diagnosis. They found that the basic social communication subgroup and RRB symptoms contributed to the prediction of an autism diagnosis, but the interaction quality subgroup did not.

Bishop et al. claimed that the basic social communication subgroup could be caused by other dysfunctions, such as hyperactivity or ID. However, as was discussed above, only if autism and ID were distinct unitary disorders with clear boundaries could ID be claimed to be the cause of autism symptoms. In fact, ID and autism are better understood as two symptom sets that occur together most often linked by gene variants, therefore it is unlikely that they are two separate disorders, one of which causes the other. Bishop et al. claimed that if ID caused basic social communication impairments, then these impairments would be poorer predictors of an autism diagnosis. Of course, the goal of transdiagnostic endophenotypes is not to predict an ASD diagnosis, nonetheless, from the Bishop et al. subgroups, *impaired social engagement*, and *poor interaction quality*, might be possible constructs.

#### The second source for possible transdiagnostic constructs is theories of the cause of ASD social impairment

Three influential theories of the cause of social impairment are unlikely to be a sound basis for the discovery of constructs for endophenotypes: (1) weak central coherence, (2) impaired Theory of Mind (ToM), and (3) impaired executive function. Bottema-Beutel et al. (66) conducted a meta-analysis of these three theories thought to index eight social skills: imitations; responding to and initiating joint attention; pretend play; executive functions; ToM; central coherence; and visual fixation to social stimuli. The three theorized models—central coherence, ToM, executive function—accounted for just a tiny amount of variance in the eight social skills. And notably, ToM explained just 4.5% of the variance in social functioning overall. The researchers (66) concluded that accepting and employing these three theories “may have led to false conclusions about the nature of ASD, the nature of social functioning more generally, and the intervention strategies that should be implemented to support individuals with ASD” (p. 164).

Similarly, the broken mirror neuron theory of autism social deficits is unlikely to be a sound source of transdiagnostic symptoms (67, 68). Heyes and Catmur (67) presented evidence that the mirror theory of social behavior has not been explanatory. And Hamilton's review of mirror system research (68) led him to conclude that here was “little evidence for a global dysfunction of the mirror system in autism” (p. 91).

In 1967 Wing et al. theorized that the core dysfunction of autism was “a lack of response to others” (34), and in 2012 Chevalier et al. proposed that the lack of social motivation to interact with others was the core dysfunction of autism (69). They argued that early childhood impairments in social attention result in poor learning of social interaction behaviors, which in turn impairs social cognitive development. Their rationale was that humans have psychological dispositions and biological mechanisms that bias them “to preferentially orient to the social world (social orienting), to seek and take pleasure in social interactions (social reward), and to work to foster and maintain social bonds (social maintaining)” (p. 231). Chevalier et al. proposed that individuals with autism lack social motivation because they are not rewarded by orienting to others or maintaining social interaction as a result of disruptions of the orbitofrontal–striatal–amygdala circuitry. The Chevalier theory suggests that *impaired social motivation* could be a basis for an endophenotype.

Hornix et al. theorized that social behavior is crucially dependent on sensory processing and multisensory cue integration of the myriad social cues exhibited by others (70). Hornix et al. proposed that autism social withdrawal was a direct result of impaired sensory processing of social cues and impaired multisensory cue integration because without sensory processing and integration, social information cannot be comprehended. In addition, McCarty and Brumback (71) found evidence that repetitive movements or stereotypies are a byproduct of the brain's attempt to use rhythmic motor commands to regulate impaired sensory processing. They argued that the brain generates compensatory motor signals to entrain abnormal rhythms in the sensory system. They theorized that compensatory motor commands cause the repeated hand movements or body movements identified as stereotypies. The researchers also proposed that attention to mechanical rhythms in the environment, such as spinning fans, could entrain the brain's dysfunctional sensory processing. If so, stereotypies may be a motor byproduct of the brain's attempt to correct sensory dysfunction. *Sensory dysfunction* could be a basis for an endophenotype.

There are many theories of oxytocin abnormalities as a cause of autism symptoms. Grattrocki and Friston (72) proposed that “a dysfunction in the oxytocin system, early in life, could account for the development of autism” (p. 411). They argued that an aberrant oxytocin system led to problems in an awareness of self and impairment in attention to social features in the behavior of others. From their model, it is possible that *impaired social attention* might be an endophenotype construct.

The PRISM project theorized that social withdrawal is a feature of many psychiatric diagnoses (21). The rationale for the PRISM project using social withdrawal as a transdiagnostic endophenotype is the evidence that social withdrawal is the product of three distinct brain networks that are theorized to govern social behavior. The first network governs social stimuli detection and processing. The second network governs social affiliation and social aversion. The third network governs social imitation and mentalizing. Because evidence indicates that social withdrawal results from all three networks (21), the PRISM project argues that therefore social withdrawal should be a central transdiagnostic endophenotype used in the discovery of possible shared pathophysiologies across many psychiatric diagnoses. The PRISM project evidence suggests that *social withdrawal* could be an important construct for a transdiagnostic endophenotype that may link autism with other neurodevelopmental disorders.

#### The third source for possible transdiagnostic constructs is autism subgrouping studies

van Rentergem et al. (73) reviewed an exhaustive set of subgrouping studies and concluded, “there is too little evidence that the observed subtypes are valid and reliable” (p. 9). The researchers argued that future subgrouping studies must pre-register hypotheses, use follow-up data to validate subgroups, and document data that falsifies or confirms subgroup validity. However, despite these meta-analysis findings, existing subgroups may nonetheless offer clues about possible endophenotypes.

Rosello et al. reviewed several subgroup studies and reported three general subgroups: (1) severe expression of all autism diagnostic symptoms; (2) moderate social impairment with few RRBs; and (3) a low level of social impairment with a high level of RRBs (51). The three levels of social impairment—severe, moderate, low—may reflect an underlying continuous distribution of social impairment. If so, the three levels of social impairment are unlikely to generate distinctive investigatory constructs of social impairment.

Sacco et al. analyzed autism symptoms, family characteristics, and biological endophenotypes, and identified four clusters (52). The clusters were: (1) circadian and sensory dysfunctions with immune abnormalities and minimal developmental delay; (2) circadian and sensory dysfunctions without immune abnormalities; (3) stereotypies; and (4) immune abnormalities, circadian and sensory dysfunctions, disruptive behaviors, and ID. Inspection of Sacco et al.'s four clusters suggests that *sensory dysfunction* may be a possible construct for a transdiagnostic endophenotype.

As described earlier, nine autism subgroups, based on neural, biological, and clinical characteristics and developmental trajectories, were discovered by The Autism Phenome Project (33). Of these nine, only two subgroups included autism symptoms: individuals with high levels of sensory dysfunction; and females with higher IQs whose autism symptoms decreased over time. The subgroup of females may identify a variant neurodevelopmental disorder, but *sensory dysfunction* is a possible endophenotype. The Autism Phenome Project's sensory dysfunction subgroup is strengthened as a possible construct candidate because three of Sacco et al.'s four subgroups included sensory dysfunction.

Harris et al. discovered three classes of social communication symptoms and two classes of RRB symptoms in a large sample of toddlers (53). The five classes shared missing or inconsistent social communication and RRBs. Kim et al. also conducted a cluster analysis of behaviors in a sample of toddlers (54). Their first two clusters were defined by significantly delayed verbal skills, and their third and fourth clusters included more severe social impairment than the first two. However, there were many commonalities across Kim et al.'s four groups: all four had consistent levels of non-verbal communication and daily living skills. Although clusters of symptoms in these two groups of toddlers identified nine possible groups, the nine clusters share overlapping symptoms such that they don't provide a clear basis for finding constructs. However, because all of Harris and colleagues' five classes shared missing social communication, consequently *missing social communication* may be a productive construct for the discovery of a transdiagnostic endophenotype.

### Summary of candidates for endophenotypes

Examining DSM-5 ASD criteria and ADOS items suggested four possible endophenotype constructs: *inability to engage socially*; *impaired socially engaged behavior*; *impaired social engagement*; and *poor interaction quality*. These can be combined to yield two constructs: *inability to engage socially*; and *poor interaction quality*. Examining theories of social impairment in autism suggested four possible endophenotype constructs: *impaired social motivation; sensory dysfunction; impaired social attention;* and *social withdrawal*. And examining autism subgrouping studies suggested two possible endophenotype constructs: *sensory dysfun*ction and *lack of social communication*.

Combining possible constructs from all three sources yields six social impairment constructs: *sensory dysfunction*; *impaired social motivation*; *impaired social attention*; *social withdrawal*; *lack of social communication*; and *poor interaction quality*. These six constructs have been determined by inspecting diagnostic criteria, causal theories, and the products of subtyping. An analytic approach to these same sources will likely discover different constructs. However, these six constructs are a reasonable place to start.

## Pro tem sketch for a future transdiagnostic endophenotype symptom screening

The search for transdiagnostic behavioral endophenotypes requires the documentation of transdiagnostic symptoms, and thus any screening tool must include a wide range of behaviors in children referred for autism and for a range of neurodevelopmental disorders.

Six construct groups for a future transdiagnostic screening tool are sketched here. One might include *sensory dysfunction*; *impaired social motivation*; and *impaired social attention*. A second construct group might include *social withdrawal* and *lack of social communication*. And a third construct might be *poor interaction quality*. A fourth group of symptoms might include ID behaviors, a fifth might include ADHD behaviors, and a sixth might include symptoms of anxiety.

Of course, as noted, these six groups are just a pro tem sketch. Only an analysis of the relationships between transdiagnostic symptoms can determine whether there are construct groups, and what they may include. Below are some possible specific symptoms that might be included in the sketched construct groups.

### The first construct group might include measures of sensory dysfunction, impaired social motivation, and impaired social attention

Possible measures for this construct group might include lack of eye contact, total lack of facial expressions, failure to express affect, and inattention to others. Other possible measures might include overly focused interest in moving objects or parts of objects, becoming upset by slight changes in a routine and having difficulty with transitions. Additional measure might include being more sensitive or less sensitive than other people to sensory input, such as light, sound, clothing, or temperature.

### The second construct group might include measures of social withdrawal and lack of social communication

Possible measures might include lack of non-verbal communication, failed joint attention, and failure to initiate or respond to social interactions.

### The third construct group might include measures of poor interaction quality

Measures of poor interaction quality might include infrequent sharing of interests, talking at length about a favorite subject without noticing others, expressing incongruous emotional displays, speaking in an odd tone, and having trouble understanding another person's point of view.

### The fourth construct group might include measures of adaptive behaviors that include items indexing cognitive functioning

Jonkers et al. tested his measure Adaptive Ability Performance Test (ADAPT) and found it to be a valid instrument for assessing difficulties in adaptive skills (74). The researchers reported that adaptive behaviors could be divided into three domains: conceptual, social, and practical. Specific items included brushing teeth; washing hands; maintaining relationships; taking the initiative to talk; thinking before acting; learning from mistakes; and the ability to stop an action if necessary. A wide array of behavior problems can also be indexed by the Child Behavior Checklist (CBCL), a questionnaire to assess behavioral and emotional problems (75). There are seven scales of symptoms for young children: emotional reactivity; anxious/depressed; somatic complaints; withdrawal; sleep problems; attention problems; and aggressive behavior. The CBCL was tested as a measure of autism (76) and it was discovered that “children with ASD had significantly higher scores than controls… (on) all syndrome scales” (p. 6).

### The fifth construct group might include measures of ADHD behaviors

Llanes et al. identified the symptoms that predict an ADHD diagnosis (77). These include the inability to concentrate, sit still, finish a project, pay attention, follow directions, and be quiet. The Connors Parents Rating Scale-Revised (78) is another source of ADHD symptoms including items such as needs supervision to get through assignments, is easily distracted, has difficulty in engaging in tasks, and is restless.

### The sixth construct group might include measures of anxiety

Muris et al. described types of anxiety in children: separation anxiety disorder; selective mutism; social anxiety disorder; panic disorder; generalized anxiety disorder; agoraphobia; and phobias (79). Muris et al. provided examples of items; I am afraid my parents will leave and never come back, I am so shy I don't speak at all, I find it scary to be with people I don't know, I feel panic, and I worry a lot, and I fear going out of my home.

### Summary of transdiagnostic symptom items

A screening tool is necessary to capture the possible neurodevelopmental symptoms that might contribute to transdiagnostic behavioral endophenotypes. Building a future transdiagnostic screening tool will require research to examine the effectiveness of the sets of symptoms. The constructs and items outlined here are a pro tem sketch of a future screening tool that must include a wide range of behaviors in children referred for autism and for a range of neurodevelopmental disorders. Six possible sets of symptoms were proposed: (1) sensory dysfunction; impaired social motivation; and impaired social attention; (2) social withdrawal and lack of social communication; (3) poor interaction quality; (4) impaired adaptive behaviors that also index cognitive impairment; (5) ADHD behaviors, and (6) symptoms of anxiety.

## Conclusion

DSM-5-TR will be released 72 years after DSM-1, during which time many diagnoses have come and gone. NIMH director Gordon offered the optimistic opinion that today's DSM-5 ASD studies were like May flowers (80). And he predicted there would be a “Summertime” of autism research 20 years from now, when autism heterogeneity then would have been thoroughly explained (80). Less optimistically, Miller proposed scrapping the DSM and replacing it with a diagnostic manual that simply documents complexity (81). But because the heterogeneity of autism symptoms and causes and comorbidities reflects a very complex web of relationships, it might be that only advanced artificial intelligence (82, 83) will be able to discover clearly defined significant subgroups with explanatory power for the creation of effective drug regimens and effective behavioral treatments.

DSM-5 ASD criteria are a paradigm. Researchers have adhered to this paradigm in building a body of knowledge about autism as a unitary entity. Although previous DSM autism criteria did not define just one autism diagnosis (22), the paradigm of autism as a single clinical entity now governs autism research. Despite existing heterogeneity, the DSM5 ASD diagnosis and autism diagnostic assessments such as the ADOS both assume that autism is a single disorder.

The most important problem for the DSM-5 ASD paradigm is that autism heterogeneity has impaired the explanatory power of the diagnosis (5, 6, 13, 15, 46–49). Wolfers et al. (15) stated that “it has not been possible to predict ASD to a degree that translated to clinical practice” (p. 25). Validated behavioral treatments have not yet been established (48, 49), and effective drug regimens have not been discovered (46, 47). Although the errant paradigm of an Earth-centered universe was maintained for 2,000 years, most paradigms are abandoned when there is evidence that the paradigm's explanatory power has failed. Clearly, it is crucial to abandon paradigms that fail to advance science and fail to improve public health. Believing in a fixed set of species blocked the discovery of evolution and consequently genetics. Maintaining belief in a failed paradigm has even cost lives. Many lives were lost through infections during the 90 years it took for all physicians to accept Semmelweis's paradigm of sepsis—that sepsis was caused by “ichor” (wound discharge) on unwashed physicians' hands (84).

Unfortunately, belief in autism as a single entity *has* caused harm (85). Drug regimens designed for autism as a single entity have yet to be discovered, and the effectiveness of behavioral treatments for all with ASD is uncertain. Importantly, in large part because there are no sufficiently effective treatments for autism as a whole, bogus “drug” regimens, dubious behavioral treatments, and unfounded beliefs have caused harm. In particular, the belief that vaccines cause autism has led to illness and even death (86).

Many researchers now begin their research papers by stating that autism is many disorders. However, their papers then go on to present research based on the paradigm of autism as a unitary disorder (10, 11). This is a common “straddle position” in the process of shifting to a new paradigm (autism is many disorders) from an old paradigm (autism is one disorder). For example, Casanova et al. (8) described autism as a group of complex conditions, but defined autism as one disorder with wide boundaries. Lai et al. (10) began their paper by stating that “Autism is a set of heterogeneous neurodevelopmental conditions” (p. 896). However, in all the sections of their paper the authors discuss autism as a unitary entity (10), making claims such as that autism has a high heritability, that the brain bases of autism have been found at the neuroanatomical level, and that more males are diagnosed with autism than females. If autism is a set of heterogeneous neurodevelopmental conditions, these varied conditions cannot have one high heritability or one neuroanatomical brain dysfunction.

This paper has proposed that autism heterogeneity stands against the paradigm that autism is a single unitary clinical entity. Although DSM-5 ASD has been shown to differ from typical development, DSM-5 ASD remains a theoretical paradigm that has not been tested as a whole (87). Transdiagnostic behavioral endophenotypes may or may not form groups with more explanatory power than the single autism diagnosis. But only when researchers test the unitary autism paradigm as an unproven theory, may new paradigms with more explanatory power be found.

## Author contributions

The author confirms being the sole contributor of this work and has approved it for publication.

## Conflict of interest

The author declares that the research was conducted in the absence of any commercial or financial relationships that could be construed as a potential conflict of interest.

## Publisher's note

All claims expressed in this article are solely those of the authors and do not necessarily represent those of their affiliated organizations, or those of the publisher, the editors and the reviewers. Any product that may be evaluated in this article, or claim that may be made by its manufacturer, is not guaranteed or endorsed by the publisher.
